# Mortality, cancer incidence, and disability among professional drivers in Slovenia

**DOI:** 10.2478/aiht-2023-74-3784

**Published:** 2023-12-29

**Authors:** Andrea Margan, Metoda Dodič Fikfak

**Affiliations:** Ljubljana University Medical Centre, Institute of Occupational, Traffic, and Sports Medicine, Ljubljana, Slovenia

**Keywords:** healthy worker effect, occupational disability, standardised cancer incidence ratio, standardised disability ratio, standardised mortality ratio, standardised proportional mortality ratio, delovna invalidnost, standardizirano proporcionalno razmerje umrljivosti, standardizirano razmerje incidence raka, standardizirano razmerje invalidnosti, standardizirano razmerje umrljivosti, učinek zdravega delavca

## Abstract

Literature data about all-cause and cause-specific mortality among professional drivers are inconsistent. Most studies report lower all-cause and higher cause-specific mortality. Higher cause-specific mortality is most often the result of malignant and circulatory diseases. The aim of our retrospective cohort study was to get a better insight into the mortality, cancer incidence, and occupational disability of the entire professional driver population in Slovenia (N=8,231) from 1997 to 2016 through standardised mortality ratio (SMR), standardised proportional mortality ratio (SPMR), standardised cancer incidence ratio (SIR), and standardised disability ratio (SDR). Total mortality was significantly lower than that of the general working population (SMR=0.49; 95 % CI=0.44–0.55). When SPMR was calculated, however, the risk of all-cause mortality increased to 1 (SPMR=1.00; 95 % CI=0.89–1.12), of cancer-related mortality to 1.13 (95 % CI=0.94–1.35), and of injury-related mortality to 1.25 (95 % CI=0.97–1.59). Cancer incidence was lower than in the general male working population for all types of cancer (SIR=0.66; 95 % CI=0.59–0.72), lung cancer included (SIR=0.56; 95 % CI=0.41–0.73). Occupational all-cause and cause-specific disability were also lower than in the rest of the working population. Even though all types of cancer and injuries were established among professional drivers in Slovenia, no major risk stand out. However, our findings may have been skewed by the healthy worker effect.

## Abstract (sl)

Podatki o splošni in specifični umrljivosti poklicnih voznikov so nekonsistentni. Večina raziskav poroča o nižji splošni in višji specifični umrljivosti poklicnih voznikov. Kot najpogostejša vzroka višje specifične umrljivosti poklicnih voznikov se omenjajo maligne bolezni in bolezni obtočil. Med malignimi boleznimi so najpogostejši malignomi dihal, sečil in prebavil, kar se povezuje tudi z izpostavljenostjo dizelskim plinom, ki so dokazani karcinogeni. Namen naše retrospektivne kohortne študije je bil pridobiti boljši vpogled v umrljivost, incidenco raka in delovno invalidnost poklicnih voznikov. V kohorto smo vključili 8231 moških voznikov, ki so bili v obdobju od 1997 do 2016 vključeni v poklicno zavarovanje. Uporabili smo indirektno standardizacijo in izračunali standardizirano razmerje umrljivosti (SMR), standardizirano proporcionalno razmerje umrljivosti (SPMR), standardizirano razmerje incidence raka (SIR) in standardizirano razmerje invalidnosti (SDR). Splošna umrljivost moških poklicnih voznikov je bila pomembno nižja kot pri splošni delovno aktivni populaciji (SMR=0,49; 95 % IZ=0,44–0,55). Pri izračunu SPMR se je tveganje za umrljivost zaradi vseh vzrokov zvišalo na 1 (SPMR=1,00; 95 % CI=0,89–1,12), za umrljivost, zaradi malignih bolezni na 1,13 (95 % CI=0,94–1,35) in zaradi poškodb na 1,25 (95 % IZ=0,97–1,59). Incidenca raka je bila v skupini poklicnih voznikov nižja kot pri delovno aktivni populaciji za vse in specifične vrste raka (SIR=0,66; 95 % IZ=0,59–0,72), vključno s pljučnim rakom (SIR=0,56; 95 % IZ=0,41–0,73). Tudi splošna in specifična delovna invalidnost je v skupini poklicnih voznikov nižja kot pri delovno aktivni populaciji. Na rezultate bi lahko vplival učinek zdravega delavca.

In Slovenia, the assessment of medical fitness of professional drivers is mandated by law (1). This means that professional drivers must meet certain health requirements before they start to work and then take regular examinations every five years. In addition, professional drivers are covered by occupational insurance and have an accelerated retirement plan; 12 months of work counts as 14 months (2).

Mortality and morbidity studies of professional drivers are relatively scarce and their results are inconsistent. Only a few report higher mortality than in the general population over cardiovascular events (3, 4, 5, 6, 7), road-traffic injuries (8), and malignant diseases, such as respiratory and gastrointestinal cancer (5, 9, 10, 11, 12, 13). Large cohort studies report higher incidence of these types of cancer among professional drivers as associated with excessive alcohol intake and smoking (14, 15, 16). Higher incidence of malignant diseases among professional drivers is also closely associated with exposure to polycyclic aromatic hydrocarbons (PAHs) in diesel engine exhaust (17, 18), even if the effect of smoking is accounted for (19). The most common types of occupational cancer reported among drivers are lung (14, 15, 16) and bladder cancer (20, 21, 22). The former has been evidenced to increase with cumulative exposure to diesel engine exhaust (23).

The aim of our study was to fill a void in information about these risks in professional drivers in Slovenia by establishing whether they had higher all-cause and cause-specific mortality, cancer incidence, and all-cause and cause-specific permanent occupational disability than the general working population.

## METHODS

This retrospective cohort study included professional drivers registered in the Database of Employees under the Accelerated Retirement Scheme (the Slovenian Pension and Disability Insurance Institute database) and the Database of Employees Covered by Compulsory Supplementary Pension Insurance (Occupational Insurance; Kapitalska Družba d.d. database) from 1997 to 2016. Since 1974, professional drivers are defined as those who drive at least 80 % of their working time or at least 60,000 km a year (24). From the 8,369 professional drivers registered in the two databases we excluded those with incomplete data, so that the study included 8,231 (98 %) drivers. Because there were only 75 professional women drivers, all further analyses included men only (N=8,156).

Data on mortality, cancer incidence, and occupational disability were taken from the national death registry (available at the National Institute of Public Health portal) (25), cancer registry (available at the Slovenian Cancer Registry portal) (26), and the national occupational disability database (kept by the Slovenian Pension and Disability Insurance Fund). In Slovenia, there are three categories of disability: the first covers complete loss of the ability to work and the second and the third cover different degrees of limited ability to work. For this study, we took into account only the first category of disability. The analysis of the cause of occupational disability only took into account the main diagnosis that led to the disability and cases that developed up to two years after the end of employment in an occupational category to cover cases developed while the person was still working but got the disability status later. If we had not limited this period to two years, we would have had disability cases associated with work in jobs other than driving.

The number of person-years was calculated for every professional driver included in the study for each year observed, considering the period in which the person worked as a professional driver, from their first job onward or from the start of the observation period (1 January 1997) onward. Person-years were calculated up to the day a change occurred (e.g., death, cancer diagnosis, or occupational disability) or until the end of the observation period (31 December 2016) for those who did not die, fall ill, or become disabled. Only malignant neoplasms were considered in this data analysis. Furthermore, for the analysis of cancer incidence we took only the first diagnoses of malignant tumours to control for the possibility that individuals diagnosed with cancer for the second or more times should be more susceptible to congenital cancer.

From the obtained mortality data (the number of total deaths and deaths by ICD-10 category) and cancer incidence data in the general male working population for each year from 1997 to 2016 we calculated the expected number of deaths and cancer cases for all causes together and by the relevant ICD-10 category. For the working population we took the general male population aged 20 to 65 years. The general male working population and professional drivers were stratified into 10 five-year age groups. The expected number of disabled individuals was calculated from the observed number of disabled male workers registered with the Slovenian Pension and Disability Insurance Fund by age group.

Based on the expected and observed numbers of deaths, cancer cases, and disabled individuals in the cohort, we calculated the total and specific standardised mortality, cancer incidence, and disability ratios for all and for specific causes for all professional drivers and by length of employment (<10 years, 10–19 years, ≥20 years), taking into account latency of 5 and 10 years (the latent period was taken into account for mortality and cancer incidence) (27, 28, 29).

When a study population is relatively healthy, such as an occupational population, epidemiological studies are likely to underestimate risk. Thus, in addition to the standardised mortality ratio (SMR), we used the standardised proportional mortality ratio (SPMR) to account for the healthy worker effect. Whereas the SPMR is also obtained by dividing the observed number of cases by the expected number of cases, the expected number of cases is calculated based on the proportion of deaths due to a specific cause among all deaths in the standard population (30), in this case the Slovenian general male working population.

### Statistical analysis

Standardised ratios and person-years were calculated using the IBM SPSS Statistics 25.0 (IBM Corp. Armonk, NY, USA) and Microsoft Office Excel 2016 (Microsoft Corp., Reston, VA, USA) software and are given as 95 % confidence intervals, taking into account the Poisson distribution (31, 32, 33).

## RESULTS

**Figure 1:**
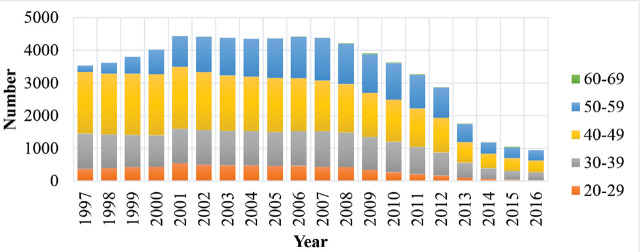
Number of male professional drivers in Slovenia between 1997 and 2016 by age group

Between 1997 and 2016, the average age of male professional drivers increased from 40.33 (in 1997) to 45.60 years (in 2016). The largest age group was 40–49 years (Figure 1).

Of the included men, 326 died between 1997 and 2016. The most common cause of death was cancer (N=125), followed by circulatory diseases (N=76) and injuries, poisoning, and other external causes (N=68) (Table 1). Gastrointestinal cancer was the most common type of cancer (N=38), followed by respiratory and intrathoracic cancer (N=35). This number of deaths is significantly lower than expected for the general male working population both in terms of all-cause mortality (Table 2) and cause-specific mortality, irrespective of the length of employment or latency.

**Table 1:** Number deaths by the International Statistical Classification of Diseases and Related Health Problems, 10^th^ revision (ICD-10) among male professional drivers in Slovenia from 1997 to 2016

**ICD-10**	**N**
Certain infectious and parasitic diseases	1
Neoplasms	125
Endocrine, nutritional and metabolic diseases	3
Mental and behavioural disorders	12
Diseases of the nervous system	3
Diseases of the circulatory system	76
Diseases of the respiratory system	5
Diseases of the digestive system	13
Symptoms, signs, and abnormal clinical and laboratory	
findings, not elsewhere classified	20
Injury, poisoning and other consequences of external causes	68
**Total**	**326**

**Table 2:** Standardised mortality ratio (SMR) among male professional drivers by length of employment and latency period (1997–2016)

	**Total**	**Employment**			**Working for at least one year**	**Latency**	
		**<10 yrs**	**10–19 yrs**	**≥20 yrs**		**5 yrs**	**10 yrs**
Expected deaths	664.79	163.83	150.08	350.87	644.75	612.26	552.57
Observed deaths	326	95	49	182	307	297	265
SMR	0.49^*^	0.58^*^	0.33^*^	0.52^*^	0.48^*^	0.49^*^	0.48^*^
95 % CI, lower limit	0.44	0.47	0.24	0.45	0.42	0.43	0.42
95 % CI, upper limit	0.55	0.71	0.43	0.60	0.53	0.54	0.54

* statistically significant difference from the general male working population

When we reanalysed the data for the SPMR, the risk of all-cause mortality increased even with the latency taken into account (Table 3). Even though the values obtained are not statistically significant, a higher risk was observed for mortality from all cancers (SPMR=1.13; 95 % CI=0.94–1.35) and from injuries, poisoning, and other external causes (SPMR=1.25; 95 % CI=0.97–1.59).

**Table 3:** Standardised proportional mortality ratio (SPMR) among male professional drivers by length of employment and latency period (1997–2016)

	**Total**	**Employment**			**Working for at least one year**	**Latency**	
		**<10 yrs**	**10–19 yrs**	**≥20 yrs**		**5 yrs**	**10 yrs**
Expected deaths	326.00	95.00	49.00	182.00	307.00	297.00	265.00
Observed deaths	326	95	49	182	307	297	265
SPMR	1.00^*^	1.00^*^	1.00^*^	1.00^*^	1.00^*^	1.00^*^	1.00^*^
95 % CI, lower limit	0.89	0.89	0.74	0.86	0.89	0.89	0.88
95 % CI, upper limit	1.12	1,22	1.32	1.16	1.11	1.12	1.13

* statistically significant difference from the general male working population

There were 397 cases of first cancer diagnosed in male professional drivers between 1997 and 2016, 29.7 % of whom died of cancer. The average age at first diagnosis was 55.7 years. The average time between first day at work and first cancer diagnosis was 26.8 years (0.2–46.1 years). The average time between the end of employment and first cancer diagnosis was 6.2 years (0.01–15.5 years). Most drivers were diagnosed with gastrointestinal cancer (23 %), followed by the male reproductive system cancer (22 %), skin, respiratory, and intrathoracic cancer (15 % each). Compared to the general male working population, the standardised cancer incidence ratio (SIR) in our male professional driver cohort is significantly lower irrespective of the length of employment or latency (Table 4). Lung and prostate cancer incidences are also significantly lower than expected in the general male working population, but colorectal and bladder cancer incidences are not significantly different (Table 5) and are expected to be higher (bladder cancer SIR=1.21; 95 % CI=0.60–2.17; colorectal cancer SIR=1.15; 95 % CI=0.73–1.72) after 20 years of professional driving.

**Table 4:** Standardised cancer incidence ratio (SIR) among male professional drivers by length of employment and latency period (1997–2016)

	**Total**	**Employment**			**Working for at least one year**	**Latency**	
		**<10 yrs**	**10–19 yrs**	**≥20 yrs**		**5 yrs**	**10 yrs**
Expected cancer incidence	610.43	143.59	127.74	339.09	590.54	570.36	516.30
Observed cancer incidence	397	81	84	232	389	368	346
SIR	0.66^*^	0.56^*^	0.66^*^	0.68^*^	0.66^*^	0.65^*^	0.67^*^
95 % CI, lower limit	0.59	0.45	0.52	0.60	0.59	0.58	0.60
95 % CI, upper limit	0.72	0.70	0.81	0.78	0.73	0.71	0.74

* statistically significant difference from the general male working population

**Table 5:** Standardised cancer incidence ratio (SIR) among male professional drivers by cancer type (1997–2016)

**Cancer type**	**Expected cancer incidence**	**Observed cancer incidence**	**SIR**	**95 % CI**
Lung cancer	91.86	51	0.56^*^	0.41–0.73
Prostate cancer	98.64	73	0.74^*^	0.58–0.93
Bladder cancer	14.96	13	0.87	0.46–1.49
Colorectal cancer	34.65	35	1.01	0.70–1.40

* statistically significant difference from the general male working population

Seventy-eight cases of occupational disability among male professional drivers were observed between 1997 and 2016, mostly due to neoplasms (N=25), circulatory diseases (N=15), and mental and behavioural disorders (N=11). The standardised disability ratio (SDR) is significantly lower for all causes of disability and by ICD-10 category (neoplasms: SDR=0.50; 95 % CI=0.32–0.74; circulatory diseases: SDR=0.29; 95 % CI=0.16–0.47) than expected in the general male working population, except for eye and endocrine diseases. A detailed analysis shows no association between length of employment and occupational disability (Table 6).

**Table 6:** Standardised disability ratio (SDR) among professional drivers (men) by length of employment (1997–2016)

	**Total**	**Employment**			**Working for at least one year**
		**<10 yrs**	**10–19 yrs**	**≥20 yrs**	
Expected disability	262.61	58.86	76.55	127.20	261.74
Observed disability	78	22	14	42	75
SDR	0.30^*^	0.37^*^	0.18^*^	0.33^*^	0.29^*^
95 % CI, lower limit	0.23	0.23	0.10	0.24	0.23
95 % CI, upper limit	0.37	0.57	0.31	0.45	0.36

* statistically significant difference from the general male working population

## DISCUSSION

Between 1997 and 2016, mortality among Slovenian male professional drivers was lower than expected for the general male working population. A higher risk of mortality is associated with neoplasms and injuries, poisoning, and external causes but not with circulatory diseases and mental illnesses. These findings are in line with reports of higher mortality of professional drivers due to injuries resulting from traffic accidents (8) and malignant diseases (5, 9, 10).

Speaking of which, the literature describes a higher risk of all types of neoplasms and specific types of cancer (lung, bladder, prostate, and gastrointestinal cancer) among professional drivers (14, 15, 34, 35), yet we have not established a higher standardised cancer incidence ratio for our cohort, regardless of employment length and latency period. However, a higher risk of bladder and colorectal cancer development can be expected after 20 years of professional driving.

Similarly, the risk of occupational disability among professional drivers was lower than among the general male working population regardless of the length of employment. However, unlike for mortality, our analysis of cancer incidence and work disability has not taken into account the healthy worker effect expected for this occupation, considering that anyone who does not meet the health requirements (1) is not allowed to work as a professional driver. If we wanted to control for the healthy worker effect, we would have to compare the incidence of cancer and work disability with more appropriate reference populations, such as firefighters or police officers.

The limitations of this study arise from its inability to cover the entire population of professional drivers in the period observed. It was impossible to obtain more accurate data than those collected from the databases used. We assume that a certain amount of taxi, truck, and bus drivers who are self-employed or have their own passenger and freight transport companies are not covered by occupational insurance and therefore most likely missing. Another reason for assuming that the cohort did not cover all drivers is that some employers avoid registering their drivers in the occupational insurance system. However, these missing drivers probably do not differ from those included in the cohort and their inclusion would not significantly affect our findings.

Another limitation of the study is that we could not obtain more detail (e.g., kilometres driven, type of vehicle driven, smoking, and psychosocial factors) beyond length of employment because of Slovenia's restrictive personal data protection law. Other studies also report limited data on these potential confounders (3, 10, 36), but we believe they are very similar for all Slovenian professional drivers and would not affect the results.

Besides the healthy worker effect, another reason for the lower health risk in our driver cohort may be improved working conditions (better suspension, ergonomically designed driver cabin, or unloading aides).

To gain a better insight into the mortality and morbidity/disability risks of professional drivers, further research should therefore try to eliminate the healthy worker effect, include psychosocial factors, and compare professional drivers to similar controlled occupations as a reference group.
